# Deflating abdominal pseudocyst causing temporary normalization of ventriculoperitoneal shunt malfunction

**DOI:** 10.12669/pjms.38.6.6278

**Published:** 2022

**Authors:** Mubarak Ali Algahtany, Zeljko Kojadinovic, Aws Algahtany

**Affiliations:** 1 Mubarak Ali Algahtany, Division of Neurosurgery, Department of Surgery, College of Medicine, King Khalid University, Abha, Saudi Arabia; 2 Zeljko Kojadinovic, Department of Neurosurgery, Asir Central Hospital, Abha, Saudi Arabia; 3 Aws Algahtany Medical Student, College of Medicine, King Khalid University, Abha, Saudi Arabia

**Keywords:** Pseudocyst, Collapse, Variable, Hydrocephalus

## Abstract

Ventriculoperitoneal shunt (VPS)-related abdominal pseudocyst (APC) is a rare cause of shunt malfunction. Variable VPS function due to APC has not been described before. A 21-year-old male with hydrocephalus and bilateral VPS presented with a right-sided shunt malfunction. After a routine abdominal ultrasound (US), that proved to be unremarkable, the patient had a clinical and radiological improvement followed by a relapse. An abdominal computed tomography scan subsequently showed an APC around the peritoneal catheter tip. Laparoscopic intervention on the APC cured the shunt malfunction. We believe that the APC emptied during the compression involved while performing the abdominal US. The pseudocyst collapse led to missing it on the abdominal US and explains the short-lived clinical and radiological improvement. We introduce the concept of APC-related variable VPS function, discuss the possible mechanisms by which the pseudocyst deflated, and make suggestions toward this diagnostic problem.

***Key Messages:*** A collapsible abdominal pseudocyst could result in a variable ventriculoperitoneal shunt function. Starting the abdominal ultrasound examination over the location of the peritoneal catheter tip may overcome the collapse. Contrasted computed tomography is superior to ultrasound in diagnosing the pseudocyst.

**Abbreviations:**
APC: Abdominal pseudocyst
CSF: Cerebrospinal fluid
CT: Computed tomography
MRI: Magnetic resonance imaging
US: Ultrasound
VPS: Ventriculoperitoneal shunt.

## INTRODUCTION

The ventriculoperitoneal shunt is the traditional treatment of symptomatic hydrocephalus. Though complications of VPS are common, abdominal pseudocysts are rare.^1^,^2^ The two main reasons implicated in the pathogenesis of APC are local inflammation, mediated by an allergic reaction of local tissues to a foreign body, and infection.^3^ The clinical presentation of APC includes symptoms of shunt malfunction, abdominal symptoms (pain, abdominal swelling), or both. This case is the first report of an abdominal pseudocyst causing variable VPS function. We describe the case, outline the possible mechanisms involved, and make a recommendation for the investigation protocol in cases of a suspected pseudocyst.

## CASE REPORT

**Fig.1:**
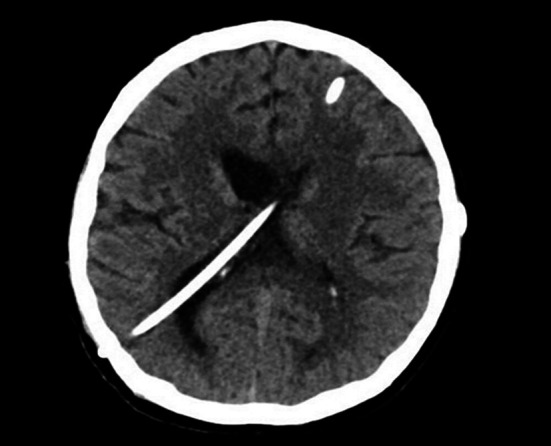
Plain CT brain scan axial image demonstrates bilateral functioning VPS.

**Fig.2:**
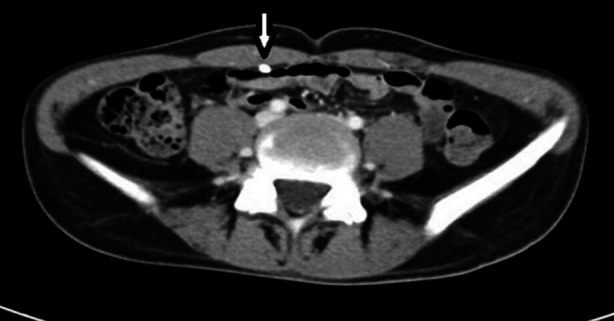
Contrast-enhanced CT abdomen scan axial image demonstrates the normal right iliac fossa two months before the patient most recent hospitalization. The white arrow points to the intraperitoneal catheter of the right-sided VPS.

A 21-year-old male developed tuberculous meningitis-related multiloculated hydrocephalus. Bilateral VPS were inserted with two consecutive right-sided proximal revisions within six months (Fig.1). The cerebrospinal fluid (CSF) obtained during surgeries did not grow any organism. A contrast-enhanced abdominal CT scan was performed immediately before the last revision and was within normal parameters (Fig.2).

**Fig.3:**
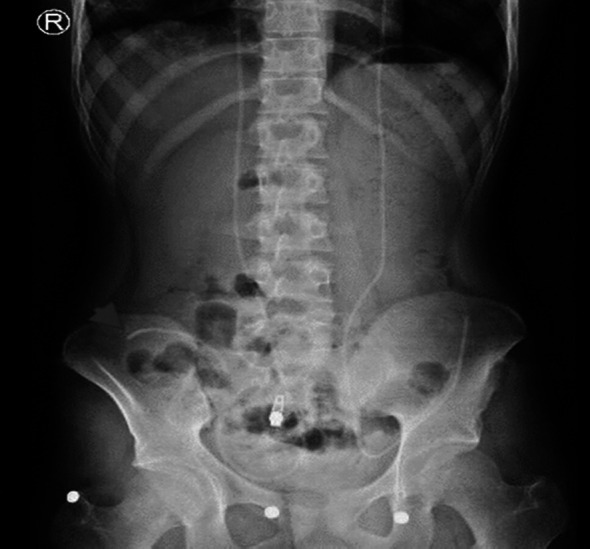
Plain AP abdomen radiograph demonstrates bilateral VPS peritoneal catheters with the tip of the right catheter in the right iliac fossa (arrowhead).

The patient presented two months later with another episode of classic symptoms of VPS malfunction but no abdominal symptoms. An X-ray of the VPS course confirmed both shunts’ continuity and showed the tip of the right VPS in the right iliac fossa (Fig.3).

**Fig.4:**
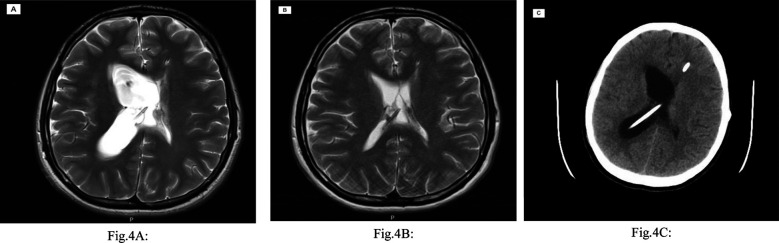
(A & B) MRI brain axial T2 images. (A) A scan obtained before the abdominal US demonstrates dilated entrapped right lateral ventricle due to right-sided VPS malfunction. (B) A scan obtained two days after the abdominal US and demonstrates improved right-sided hydrocephalus. (C) A Plain CT brain scan axial image obtained seven days after abdominal US demonstrates recurrent hydrocephalus related to right-sided VPS malfunction.

A brain MRI demonstrated evidence of right VPS malfunction (Fig.4A). An abdominal ultrasound was performed, given the recent proximal shunt revision, and yielded normal results. The patient, however, improved shortly after the ultrasound procedure. A follow-up brain MRI also demonstrated improved right-sided hydrocephalus (Fig.4B). The presenting symptoms, however, recurred within five days of in-hospital observation and a follow-up brain CT scan showed recurrence of the right-sided hydrocephalus (Fig.4C).

**Fig.5:**
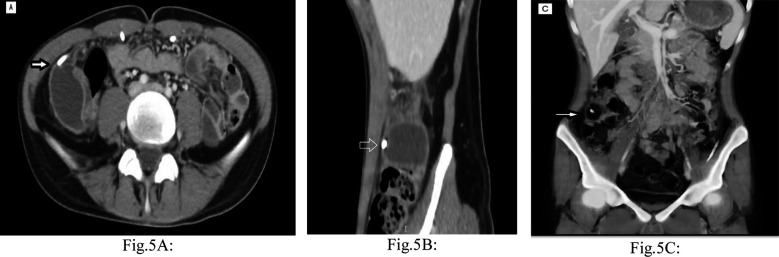
Contrast-enhanced CT abdomen scan (A) axial, (B) sagittal, and (C) coronal 3D reconstruction images demonstrate APC in the right iliac fossa with the tip of the right-sided VPS peritoneal catheter barely inside it (white arrows).

**Fig.6:**
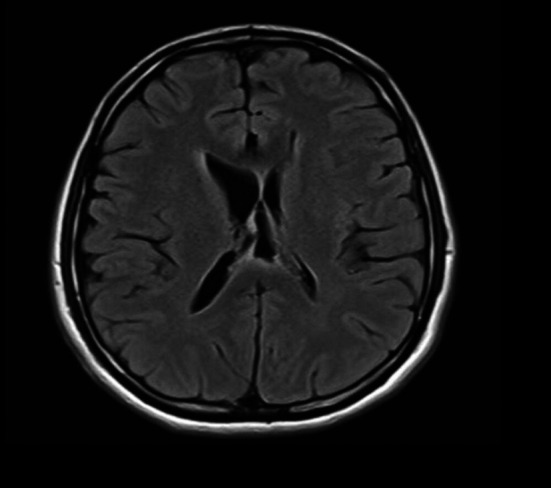
MRI brain scan axial T1 image demonstrates resolved hydrocephalus at 6 months follow up post APC resection.

An abdominal contrast-enhanced CT scan was then performed and revealed a small APC in the right iliac fossa with the tip of the right peritoneal catheter barely inside it (Fig.5A-C). The pseudocyst was treated by laparoscopic resection of its walls and liberation of the involved convolutions of the ileum. The peritoneal catheter was repositioned in another abdominal quadrant. The content of the pseudocyst was the normal CSF. The cultures of CSF and pseudocyst tissue were negative, and the histopathological analysis showed inflammatory changes. Following surgery, the patient’s symptoms resolved and remained so at the last follow-up, six months later. A brain MRI obtained at six months visit showed no evidence of recurrent hydrocephalus (Fig.6).

## DISCUSSION

The abdominal pseudocyst is a rare complication of VPS with an incidence of 1- 4.5%.^3^-^5^ This case sheds light on some of the elusive aspects of the clinical entity of APC. A brief period of clinical and radiological improvement of VPS malfunction occurred after the abdominal ultrasound procedure. Shortly, clinical and radiological signs of VPS malfunction recurred, and a small VPS-related APC emerged on the abdominal CT scan.

The raised pressure within the APC interferes with optimal VPS function. The egress of CSF from the pseudocyst, to the low-pressure abdominal cavity, temporarily normalizes the pressure and accordingly restores the shunt function. Our theory is that the APC emptied during the initial phase of the US examination owing to the pressure applied by the US probe. The pseudocyst then slowly refilled within days, causing the recurrent symptoms. We considered two possible mechanisms of temporarily emptying the APC in response to external pressure. First, CSF could leak out of the APC around the catheter entrance. The CT scan demonstration of the tip of the catheter just barely in the wall of the pseudocyst supports this presumption.

The second possible mechanism is a pseudocyst wall rupture during compression, followed by repair within days. The walls of the pseudocysts are initially made of thin fibrous tissues and the serosa of adjacent organs.^5^,^6^ In our case, the pseudocyst is still in its early formation phase (a maximum of 66 days, which is the time between the two abdominal CTs). The walls, therefore, are amenable to break with pseudocyst compression. The APCs have been shown to develop within weeks of VPS insertion and to recur within days after surgical intervention.^6^,^7^

The consequence of the pseudocyst collapse is an improvement that can mislead to early discharge of the patient. The improvement, however, is short-lived as the symptoms relapse with pseudocyst recurrence similar to the situation following APC percutaneous aspiration.^4^ Another consequence of the pseudocyst collapse is overlooking it on the ultrasound and hence misguiding the surgeon toward a proximal shunt revision.

The ultrasound has been used as the primary diagnostic tool of APC.^1^,^3^ The inability of the ultrasound to diagnose APC, distinguish it from other ascitic fluid, or to identify the end of the shunt within the pseudocyst has been documented.^5^,^6^ The ultrasound was incapable of diagnosing the small APC in this case, possibly due to its collapse upon the initial phase of the ultrasound examination. Hence suggest that the ultrasound radiographer starts the assessment over the area of the peritoneal catheter tip, as indicated by the plain radiographs. Such in this case, APC of variable sizes can be present without abdominal symptoms and maybe underdiagnosed.^1^,^3^,^5^ Contrast-enhanced CT scan is superior to ultrasound, as shown here, and is the investigative tool of choice in problematic cases.^6^-^8^ Abdominal CT scan should be performed whenever the ultrasound fails to diagnose APC in the presence of suspicion. Nevertheless, its routine use over US cannot be recommended due to the radiation involved.

## CONCLUSION

Abdominal pseudocyst can form in a matter of weeks following VPS insertion and is a well-established cause of shunt malfunction. The possibility of pseudocyst collapse due to external pressure should be kept in mind, especially in its early formation phase. Starting the US examination over the area of the peritoneal catheter tip, as indicated by the plain radiographs, may minimize this risk. The fluctuation of the patient’s symptoms, especially the improvement following diagnostic maneuvers exerting abdominal pressure, should alert to the possibility of collapsible APC. A contrast-enhanced abdominal CT scan should be performed in cases where ultrasound fails to diagnose the APC despite high suspicion.

### Authors’ Contribution:

**MAA:** Supervision, resources, writing, editing.

**ZK:** Conceptualization, writing, editing.

**AA:** Data curation, writing the original draft.

## References

[1] Hamid R, Baba AA, Bhat NA, Mufti G, Mir YA, Sajad W (2017). Post ventriculoperitoneal shunt abdominal pseudocyst:Challenges posed in management. Asian J Neurosurg.

[2] Kashyap S, Ghanchi H, Minasian T, Dong F, Miulli D (2017). Abdominal pseudocyst as a complication of ventriculoperitoneal shunt placement:Review of the literature and a proposed algorithm for treatment using 4 illustrative cases. Surg Neurol Int.

[3] Roitberg BZ, Tomita T, McLone DG (1998). Abdominal cerebrospinal fluid pseudocyst:A complication of ventriculoperitoneal shunt in children. Pediatr Neurosurg.

[4] Aparici-Robles F, Molina-Fabrega R (2008). Abdominal cerebrospinal fluid pseudocyst:A complication of ventriculoperitoneal shunts in adults. J Med Imaging Radiat Oncol.

[5] Besson R, Hladky JP, Dhellemmes P, Debeugny P (1995). Peritoneal pseudocyst-ventriculo-peritoneal shunt complications. Eur J Pediatr Surg.

[6] Erşahin Y, Mutluer S, Tekeli G (1996). Abdominal cerebrospinal fluid pseudocysts. Childs Nerv Syst.

[7] Tamura A, Shida D, Tsutsumi K (2013). Abdominal cerebrospinal fluid pseudocyst occurring 21 years after ventriculoperitoneal shunt placement:A Case Report. BMC Surg.

[8] Guest BJ, Merjanian MH, Chiu EF, Canders CP (2019). Abdominal Cerebrospinal Fluid Pseudocyst Diagnosed with Point-of-care Ultrasound. Clin Pract Cases Emerg Med.

